# Defining the protein and lipid constituents of tubular recycling endosomes

**DOI:** 10.1074/jbc.RA120.015992

**Published:** 2021-01-28

**Authors:** Trey Farmer, Shuwei Xie, Naava Naslavsky, Jacqueline Stöckli, David E. James, Steve Caplan

**Affiliations:** 1 Department of Biochemistry and Molecular Biology, University of Nebraska Medical Center, Omaha, Nebraska, USA; 2 Charles Perkins Centre, School of Life and Environmental Sciences, Sydney Medical School, The University of Sydney, Sydney, New South Wales, Australia; 3 Fred and Pamela Buffett Cancer Center, University of Nebraska Medical Center, Omaha, Nebraska, USA

**Keywords:** Rab10, MICAL-L1, EHBP1, recycling endosome, Syndapin2, DMSO, dimethyl sulfoxide, ERC, endocytic recycling compartment, FBS, fetal bovine serum, HRP, horseradish peroxidase, MEF, mouse embryonic fibroblast, PA, phosphatidic acid, PBST, PBS with 0.3% Tween, PI(4,5)P2, phosphatidylinositol-4,5-bisphosphate, PLD, phospholipase D, SE, sorting endosome, SIM, structured illumination microscopy, TRE, tubular recycling endosomes

## Abstract

Once internalized, receptors reach the sorting endosome and are either targeted for degradation or recycled to the plasma membrane, a process mediated at least in part by tubular recycling endosomes (TREs). TREs may be efficient for sorting owing to the ratio of large surface membrane area to luminal volume; following receptor segregation, TRE fission likely releases receptor-laden tubules and vesicles for recycling. Despite the importance of TRE networks for recycling, these unique structures remain poorly understood, and unresolved questions relate to their lipid and protein composition and biogenesis. Our previous studies have depicted the endocytic protein MICAL-L1 as an essential TRE constituent, and newer studies show a similar localization for the GTP-binding protein Rab10. We demonstrate that TREs are enriched in both phosphatidic acid (PA) and phosphatidylinositol-4,5-bisphosphate (PI(4,5)P2), supporting the idea of MICAL-L1 recruitment by PA and Rab10 recruitment via PI(4,5)P2. Using siRNA knock-down, we demonstrate that Rab10-marked TREs remain prominent in cells upon MICAL-L1 or Syndapin2 depletion. However, depletion of Rab10 or its interaction partner, EHBP1, led to loss of MICAL-L1-marked TREs. We next used phospholipase D inhibitors to decrease PA synthesis, acutely disrupt TREs, and enable monitoring of TRE regeneration after inhibitor washout. Rab10 depletion prevented TRE regeneration, whereas MICAL-L1 knock-down did not. It is surprising that EHBP1 depletion did not affect TRE regeneration under these conditions. Overall, our study supports a primary role for Rab10 and the requirement for PA and PI(4,5)P2 in TRE biogenesis and regeneration, with Rab10 likely linking the sorting endosome to motor proteins and the microtubule network.

The internalization of receptors and lipids from the plasma membrane is an essential process in all mammalian cells (1). Once internalized, receptor-laden vesicles are cleaved from the plasma membrane and subsequently undergo fusion with early/sorting endosomes (SEs), a crucial sorting organelle that either directs cargo to the degradation or to recycling pathways (2). Although the mechanism of targeting receptors for recycling appears to be an active process based on the recognition of receptor tails by specific proteins (3, 4, 5, 6), the actual transport pathways for recycling cargo remain incompletely understood. For example, the involvement of Rab proteins such as Rab4 (7), Rab11 (8), Rab22 (9), and other small GTP-binding proteins such as Arf6 (10) and their effectors has been well documented, but how they coordinate recycling is less well defined.

One of the mechanisms proposed for efficient endosomal sorting is the use of a large surface membrane area to segregate select molecules and target them to distinct pathways (11). Indeed, evidence suggests that some endosomal regulatory proteins serve as membrane-binding units with the propensity to induce membrane curvature and bend membranes into tubular-shaped structures (12, 13). Indeed, tubular endosomes have been observed using a variety of endosomal protein markers, including the retromer (14) and the scaffolding tubular recycling endosome (TRE) protein, MICAL-L1 (15, 16, 17, 18, 19). Moreover, MICAL-L1 interacts with both EHD1 and Syndapin2, a BAR-domain protein that is involved in membrane sculpting (20, 21, 22, 23, 24). These proteins localize to an array of TREs that transport a variety of receptors back to the plasma membrane.

TREs containing MICAL-L1 have been implicated in the trafficking of receptors both from the SE toward the perinuclear endocytic recycling compartment (ERC) and from the ERC toward the plasma membrane (25). However, the composition, structure, and mechanisms of biogenesis of these membrane compartments remain only partly known. For example, MICAL-L1-containing TREs are enriched in phosphatidic acid (PA), and there is evidence suggesting that both MICAL-L1 and Syndapin2 may be involved in their biogenesis (23). Indeed, studies with purified liposomes have shown that Syndapin2 is capable of inducing membrane tubulation, preferentially in PA-containing liposomes (23). However, a limitation of these studies was the small number of proteins that serve as markers for the TRE; accordingly, with only MICAL-L1 and Syndapin2 serving as key TRE markers, it was not possible to determine if TREs can be generated in their absence.

Recently, Rab10 has also been identified as a component of tubular endosomes (26). Indeed, the authors provide support for the localization of Rab10, at least in part, to TREs that contain MICAL-L1, and the involvement of the KIF13A/B motor proteins in TRE extension/biogenesis (26). In this article, we attempt to integrate the recent findings of Etoh and Fukuda (26) with those from our own laboratory, propose a modified and unified model for TRE composition and biogenesis, and enhance our understanding of the mechanisms by which TRE proteins function in the generation of these unique structures. We provide evidence that MICAL-L1- and Rab10-containing TREs are largely the same structures and that they are enriched in both PA and phosphatidylinositol (4, 5) bisphosphate (PI(4,5)P2). With the inclusion of Rab10 as a TRE component (26) we find that its effector EHBP1, another EHD1 interaction partner (27), is also a resident of TREs. Indeed, knock-down of either MICAL-L1 or Syndapin2 had little effect on the ability of Rab10 to localize to and/or generate TREs, whereas knock-down of either Rab10 or EHBP1 significantly impaired the generation of MICAL-L1/Syndapin2-containing TREs highlighting the importance of Rab10 in this process. Finally, we used a unique in-cell TRE regeneration assay to demonstrate that Rab10 knock-down prevented regeneration of TREs, whereas EHBP1 knock-down did not do so. Our data suggest that, although Rab10 may connect KIF13A/B motor proteins and microtubules to endosomes through an interaction with EHBP1, Rab10 may also interact with additional endosomal partners or, alternatively, interact directly with endosomal membranes via prenylation to mediate endosome tubulation.

## Results

**Figure 1:**
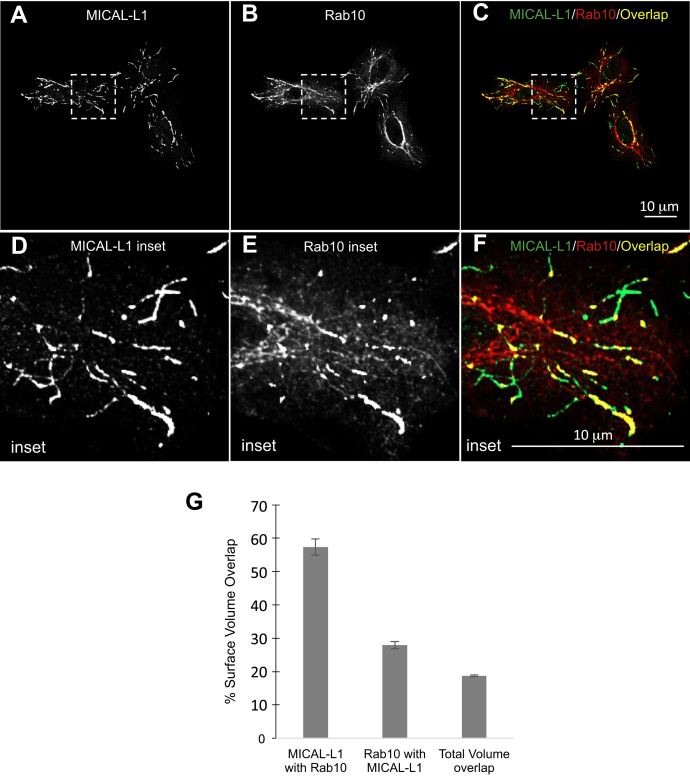
**MICAL-L1 and Rab10 are residents on the same tubular recycling endosome.***A–C*, HeLa cells were cultured on cover slides, fixed, and immunostained with antibodies against endogenous MICAL-L1 (*A*, *green*) and Rab10 (*B*, *red*). A series of serial sections were obtained and the representative image is a snapshot from a 3D reconstitution. *C*, depiction of the merged image from *A* and *B*, showing the overlap between MICAL-L1 and Rab10 in *yellow*. *D–F*, the *dashed boxes* from *A–C* are shown as magnified insets in the zoomed regions depicted in *D–F*. *G*, the graph represents three independent experiments with at least 10 images each that were subjected to imaging and quantification of surface volume overlap. The scale bars represent 10 μm. Error bars denote standard deviation.

Cells have vast arrays of tubular endosomal networks, but there is a minimal understanding of their composition, the degree to which these TRE overlap with one another, and their mechanism of biogenesis. Given the recent study suggesting potential overlap between TREs containing Rab10 and TREs containing MICAL-L1 (26), we sought to determine whether these two proteins mark the same tubular endosomes in a quantifiable manner. To address this, we immunostained fixed HeLa cells with antibodies directed against endogenous Rab10 and MICAL-L1, collected serial sections of multiple fields of cells, and used Imaris software to measure the surface volume of each protein and determine the degree to which they overlapped. Representative images and insets are shown in Fig. 1, *A*–*F*. By performing 3D surface volume analysis, we were able to avoid interference from non–membrane bound protein and focus almost exclusively on the surface volume of the endosomal proteins. In addition, the serial sections ensured that we measured true overlap and not merely proteins that overlapped in the X-Y axis (see Movie S1; Fig. S1). As demonstrated, the overall surface volume overlap of MICAL-L1 with Rab10 reached nearly 60%, strongly suggesting that MICAL-L1 and Rab10 mark the same TREs (Fig. 1*G*). The Rab10 overlap with MICAL-L1 was considerably lower, likely resulting from the more intense overall Rab10 staining pattern that includes nontubular endosomes in addition to the TREs. Even the total volume overlap, which measures the total surface volume of each protein and the percentage of overlap, was about 20%, further highlighting the degree of overlap on TREs. Moreover, overlap between MICAL-L1 and Rab10 was further validated using super-resolution Structured Illumination Microscopy (Fig. S2). Overall, our data support the notion that Rab10 and MICAL-L1 coexist on the same array of TRE.

**Figure 2:**
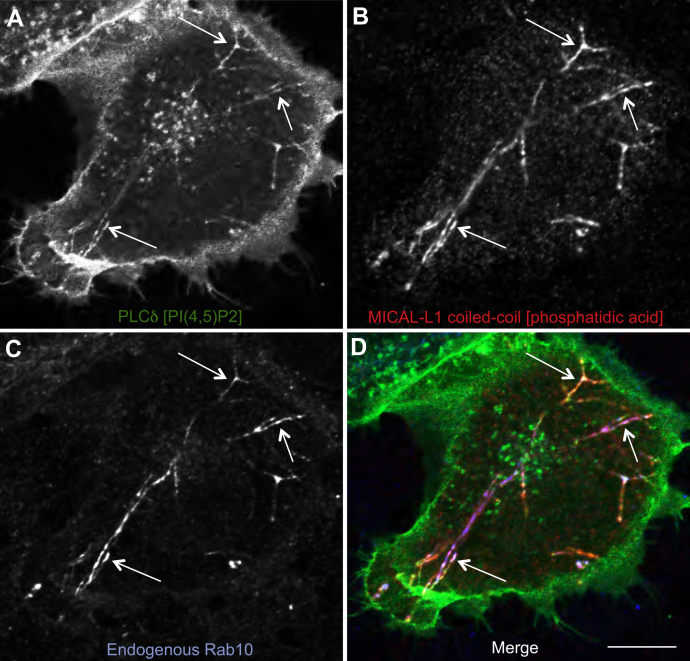
**TREs containing MICAL-L1 and Rab10 are enriched in phosphatidylinositol 4,5 bisphosphate and phosphatidic acid.***A*–*D*, HeLa cells were cotransfected with PLCδ (a marker for phosphatidylinositol 4,5 bisphosphate) and the MICAL-L1 C-terminal coiled-coil region (a marker for phosphatidic acid and TREs). Cells on coverslips were then fixed and immunostained to detect PLCδ (*A*, *green*), the MICAL-L1 C-terminal coiled-coil region (*B*, *red*), and endogenous Rab10 (*C*, *blue*). The merged three-channel image is shown in *D*. *Arrows* mark regions along TREs that are positive for endogenous Rab10 as well as phosphatidylinositol 4,5 bisphosphate and phosphatidic acid. The scale bar represents 10 μm. TRE, tubular recycling endosome.

Given the requirement for PA in recruiting MICAL-L1 to TREs (23), and the relationship between Rab10 and PI(4,5)P2 levels on endosomes, we next asked whether PA and PI(4,5)P2 are both found on Rab10/MICAL-L1 TREs. To this aim, we used the well-documented PLCδ as a well-established marker for PI(4,5)P2 (28, 29) and the MICAL-L1 C-terminal coiled-coil region as a marker for PA (23). The MICAL-L1 coiled-coil region is highly specific for PA and can be used as probe both for microscopy and *in vitro* (23). As demonstrated in Fig. 2, PLCδ localized to tubular endosomes, in addition to its localization on the plasma membrane and a series of internal vesicles that concentrated near the perinuclear region (Fig. 2*A*). The PA marker also localized primarily to long, tubular structures, but unlike PLCδ was largely devoid of localization to the plasma membrane and to perinuclear spherical vesicles (Fig. 2*B*). The localization of endogenous Rab10 closely mirrored that of the PA marker (Fig. 2*C*) and similarly was absent on the plasma membrane and perinuclear spherical vesicles. Significantly, endogenous Rab10 was found on TREs that were marked by both PI(4,5)P2 and PA (Merge, Fig. 2*D*; see arrows in Fig. 2*A–D*), indicating that these phospholipids are both enriched on TRE.

**Figure 3:**
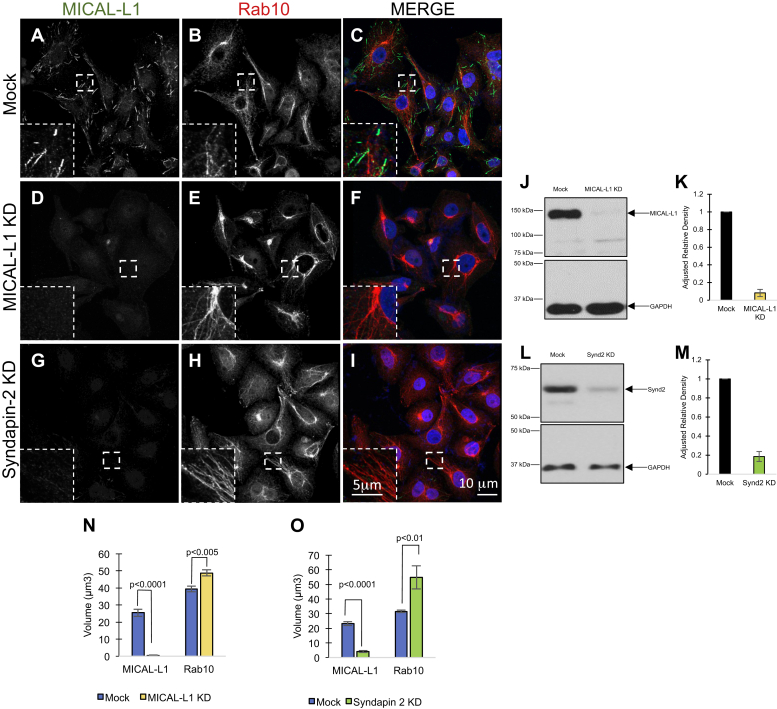
**Rab10-marked tubular recycling endosomes are not impaired in the absence of MICAL-L1 or Syndapin2.***A–I*, HeLa cells on coverslips were either Mock treated (*A–C*) or treated with either MICAL-L1 siRNA (*D–F*) or Syndapin2 siRNA (*G–I*) prior to fixation and immunostained with antibodies directed against endogenous MICAL-L1 (*A*, *D*, *G*) or endogenous Rab10 (*B*, *E*, *H*). Dual-channel merged images are shown in *C*, *F*, and *I*. *J–M*, immunoblots and quantification depicting knock-down efficiency for MICAL-L1 (*J*, *K*) and Syndapin2 (*L*, *M*). *N–O*, the mean apparent tubular recycling endosome volume was measured for MICAL-L1- and Rab10-containing structures, using Imaris software. Serial z-section imaging was done on random fields of cells on the coverslip, and pixel values were converted into surfaces using a preset threshold (see Experimental procedures). The total volume per field was calculated and then divided by the number of cells per field to derive the volume per cell for each fluorescent channel. Equivalent thresholds were used for WT and knock-down (KD) cells. At least ten fields of cells were measured and quantified in each experiment, and graphs are derived from three independent experiments. Error bars denote standard deviation, and *p*-values are derived from one-tailed Student's *t*-tests. The scale bar represents 10 μm (*inset*; 5 μm).

To determine the requirement for MICAL-L1 and Syndapin2 in Rab10-TRE generation, we measured the apparent volume of TREs marked by Rab10 (or MICAL-L1) upon MICAL-L1 or Syndapin2 knock-down. As anticipated, in Mock-treated cells, MICAL-L1 and Rab10 displayed a partial overlap on the same TREs (Fig. 3, *A*–*C*). When MICAL-L1 was depleted by siRNA (see immunoblot in Fig. 3*J*; quantified in Fig. 3*K*), the apparent volume of Rab10 TREs was not decreased (Fig. 3, *D*–*F*; quantified inFig. 3*N*). Indeed, the apparent volume of Rab10 TREs displayed a small but significant increase in the absence of MICAL-L1 (Fig. 3*N*). Since Syndapin2 interacts with MICAL-L1 and colocalizes with it on TREs, we similarly knocked down Syndapin2 and measured the apparent Rab10 and MICAL-L1 TRE volume. As demonstrated, Syndapin2 depletion led to reduced overall expression of MICAL-L1 (Fig. 3, *G–I*; knock-down validated by immunoblot in Fig. 3, *L–M*). As a result, significantly fewer MICAL-L1 TREs were measured (Fig. 3*O*), likely owing to the instability of MICAL-L1 on membranes and its degradation (23). However, Rab10 TREs were not decreased in apparent volume, and similar to MICAL-L1 knock-down, depletion of Syndapin2 significantly increased apparent Rab10 TRE volume. These data suggest that MICAL-L1 and Syndapin2 are not required for Rab10 TRE generation and/or stability but may instead play a role in TRE fission, possibly through their interaction with EHD1 (23).

**Figure 4:**
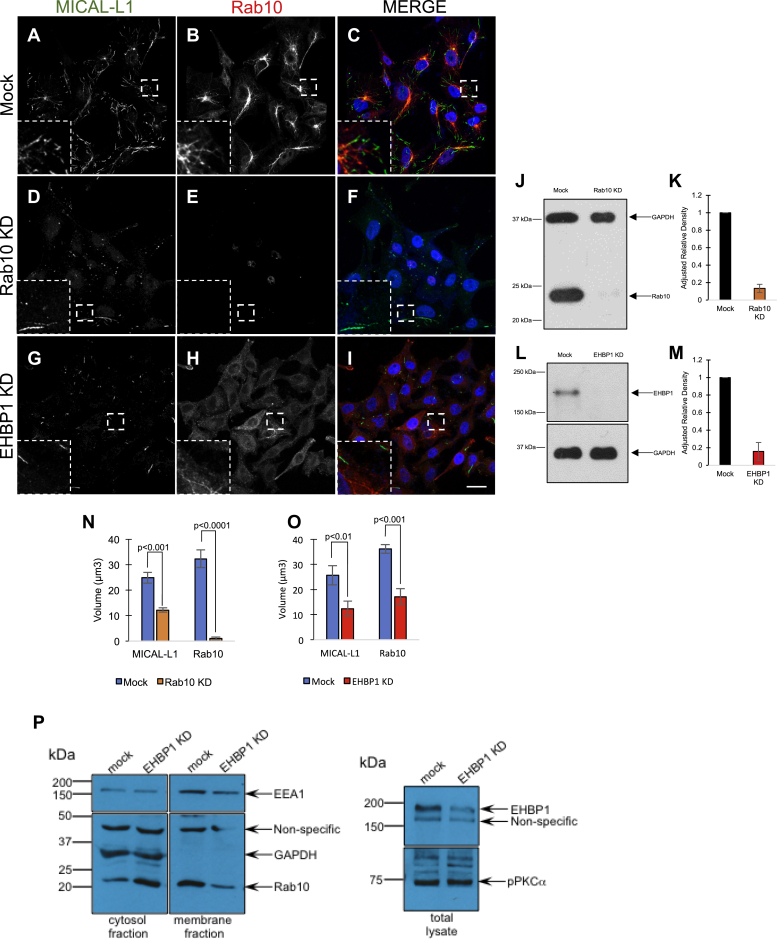
**MICAL-L1-marked tubular recycling endosomes are impaired in the absence of either Rab10 or its effector, EHBP1.***A*–*I*, HeLa cells on coverslips were either Mock treated (*A–C*), or treated with either Rab10 siRNA (*D–F*) or EHBP1 siRNA (*G–I*) prior to fixation and immunostaining with antibodies directed against endogenous MICAL-L1 (*A*, *D*, *G*) or endogenous Rab10 (*B*, *E*, *H*). Dual-channel merged images are shown in *C*, *F*, and *I*. *J–M*, immunoblots and quantification depicting knock-down efficiency for Rab10 (*J, K*) and EHBP1 (*L*, *M*). *N–O*, the mean apparent TRE volume was measured for MICAL-L1- and Rab10-containing structures, using Imaris software. Serial z-section imaging was done on random fields of cells on the coverslip, and pixel values were converted into surfaces using a preset threshold (see Experimental procedures). The total volume per field was calculated and then divided by the number of cells per field to derive the volume per cell for each fluorescent channel. Equivalent thresholds were used for WT and KD cells. At least ten fields of cells were measured and quantified in each experiment, and graphs are derived from three independent experiments. Error bars denote standard deviation, and *p*-values are derived from one-tailed Student's *t*-tests. *P*, HeLa cells were either mock treated or treated with EHBP1 siRNA. Knock-down efficiency and specificity are shown in the *right panel* (EHPB1 total lysate in mock *versus* knock-down). Mock and knock-down cells were also lysed and subjected to fractionation to membrane and cytosolic fractions, and Rab10 levels were detected by immunoblotting (*right panel*). Controls include GAPDH as an exclusively cytosolic protein and EEA1 as a primarily membrane-associated protein. The experiment displayed is a representative one from six individual experiments showing a similar trend (although the ratios of cytosolic to membrane proteins varies from experiment to experiment). The scale bar represents 10 μm (inset, 5 μm). KD, knock-down.

Given the surprisingly superfluous requirement for MICAL-L1 and Syndapin2 expression to maintain Rab10-containing TRE, we next asked whether Rab10 is required for the generation of MICAL-L1-containing TREs (Fig. 4). To this aim, we first used siRNA to knock down Rab10 expression (Fig. 4*J*; quantified in Fig. 4*K*). Compared with Mock-treated cells (Fig. 4, *A*–*C*), Rab10 knock-down cells (Fig. 4, *D*–*F*) displayed a significantly decreased apparent volume of MICAL-L1-containing TRE (quantified in Fig. 4*N*). Since Rab10 functions with EHBP1, a protein that interacts with PI(4,5)P2 and reportedly affiliates with TREs (30, 31), we knocked down EHBP1 and asked whether it impacts both Rab10- and MICAL-L1-containing TREs. As demonstrated, EHBP1 was efficiently knocked down by siRNA (Fig. 4*L*; quantified in Fig. 4*M*). When the localizations of Rab10 and MICAL-L1 were analyzed, significantly reduced Rab10- and MICAL-L1-TRE were observed, with Rab10 displaying an apparently more cytoplasmic localization (Fig. 4, *G–I*; quantified in Fig. 4*O*). Indeed, membrane fractionation experiments demonstrated that significantly more Rab10 could be detected in the cytosolic fraction and less Rab10 was detected in the membrane fraction when EHBP1 expression was impaired by EHBP1 siRNA knock-down (Fig. 4*P*). These data suggest that both Rab10 and EHBP1 are required for TRE generation and/or maintenance.

**Figure 5:**
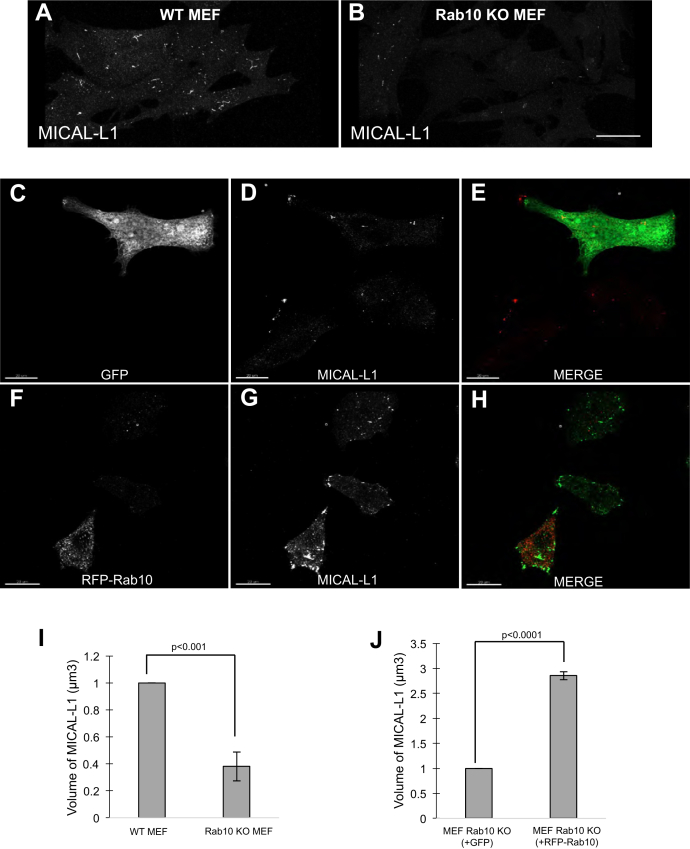
**Introduction of WT Rab10 to knockout MEFs increases the volume of MICAL-L1-containing TRE.***A*, *B*, WT MEF cells (*A*) or Rab10 KO MEF cells (*B*) were plated on coverslips, fixed, immunostained with antibodies to MICAL-L1, and imaged by confocal microscopy. Representative images from three separate experiments are depicted. *C–H*, Rab10 KO MEF cells were plated on coverslips and transfected with either GFP (control; *C–E*) or RFP-Rab10 (*F–H*). After 24 h, cells were fixed and immunostained with antibodies to detect MICAL-L1. *I*, *J*, (*I*) WT MEF and Rab10 KO MEF cells or (*J*) transfected MEF Rab10 KO cells (transfected with GFP or RFP-Rab10) were imaged by serial z-sections, and the mean apparent TRE volume was measured for MICAL-L1- and Rab10-containing structures, using Imaris software. Pixel values were converted into surfaces using a preset threshold (see Experimental procedures). The total volume per field was calculated and then divided by the number of cells per field to derive the volume per cell for each fluorescent channel. At least 10 fields of cells were measured and quantified in each experiment, and graphs are derived from three independent experiments. Error bars denote standard deviation, and *p*-values are derived from one-tailed Student's *t*-tests. The scale bars represent 10 μm. KO, knockout; MEF, mouse embryonic fibroblast; TRE, tubular recycling endosome. GFP, green fluorescent protein; RFP, red fluorescent protein.

To further address the role of Rab10 in TRE biogenesis, we examined MICAL-L1-containing TRE in mouse embryonic fibroblast (MEF) cells derived from Rab10 knockout (KO) embryos (32). Compared with wildtype (WT) MEF cells (Fig. 5*A*; quantified in Fig. 5*I*), significantly fewer TREs were observed in Rab10 KO MEF cells (Fig. 5*B*; quantified in Fig. 5*I*). Indeed, the total apparent tubular volume of MICAL-L1-marked TRE was typically two- to threefold higher in WT MEF cells than in their MEF Rab10 KO counterparts (Fig. 5*I*). Significantly, when the Rab10 KO MEF cells were “rescued” by transfection of WT Rab10 (+red fluorescent protein-Rab10) into these cells as opposed to a green fluorescent protein control, the apparent volume of MICAL-L1-marked TRE increased by ∼ three-fold (Fig. 5, *C*–*H*; quantified in Fig. 5*J*). Overall, these data support the notion that Rab10 is a major protein involved in the generation of TRE.

**Figure 6:**
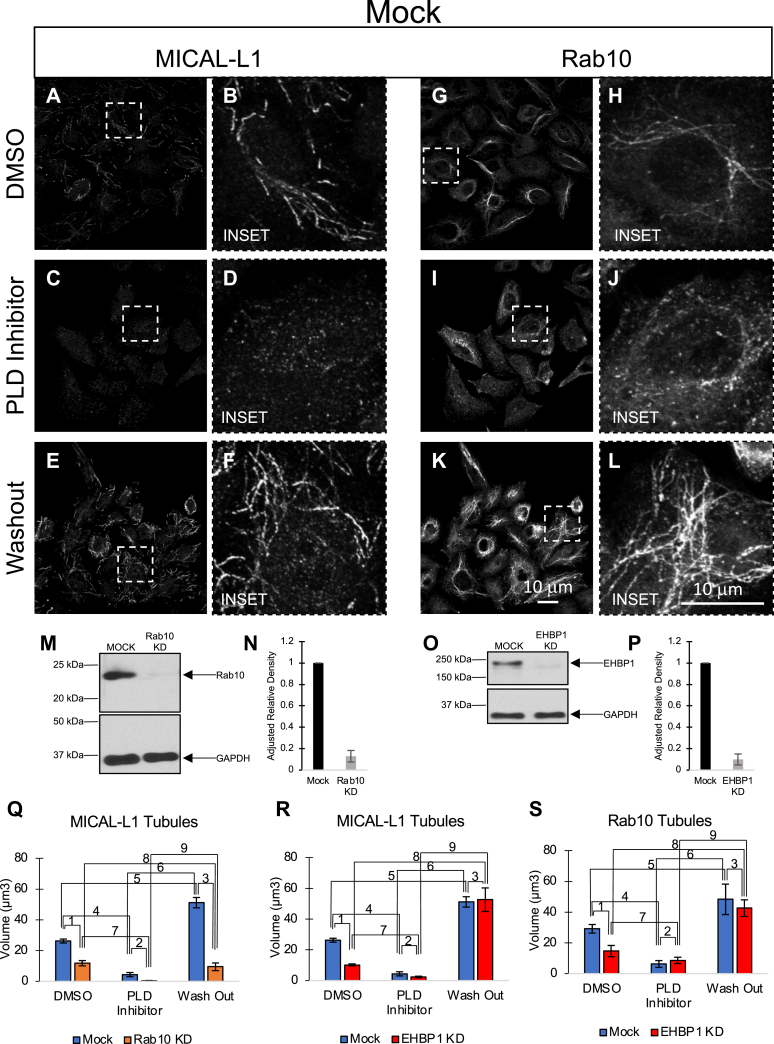
**Rab10 is a crucial protein for TRE biogenesis.***A*–*L*, Mock-treated HeLa cells were dimethyl sulfoxide (DMSO)-treated (*A*, *B*, *G*, *H*), treated with a phospholipase D (PLD) inhibitor (*C*, *D*, *I*, *J*), or treated with a PLD inhibitor followed by a washout of the inhibitor (*E*, *F*, *K*, *L*). Cells were then fixed and immunostained with antibodies directed against MICAL-L1 (*A–F*) or Rab10 (*G–L*). Enlarged insets are depicted in *B*, *D*, *F* and *H*, *J*, *L*. *M–P*, immunoblotting (*M*) and quantification (*N*) of Rab10 knock-down by siRNA and immunoblotting (*O*) and quantification (*P*) of EHBP1 knock-down by siRNA. *Q*–*S*, in addition to Mock-treated HeLa cells, HeLa cells treated with Rab10 siRNA or EHBP1 siRNA were similarly subjected to DMSO treatment, PLD inhibitors, or PLD inhibitors followed by inhibitor washout prior to fixation and immunostaining with MICAL-L1 (*Q*, R) and Rab10 (*S*) antibodies. Volumetric analysis was done as described in Figures 3 and 4. Serial z-sections were obtained by confocal imaging, and Imaris software was used to compare the mean volume of MICAL-L1 tubular recycling endosome (TRE) in mock-treated cells upon Rab10 knock-down (*Q*) or upon EHBP1 knock-down (*R*), or to compare the mean volume of Rab10 TRE in Mock-treated and EHBP1 knock-down cells (*S*). Each graph shows the TRE volume for mock and knock-down cells with DMSO treatment, PLD inhibitor treatment, and following PLD inhibitor washout. The scale bars represent 10 μm. Graphs are based on three independent experiments measuring at least five fields of cells per experiment. *p*-Values are derived from one-tailed Student's *t*-tests and are as follows: for MICAL-L1 TRE in Rab10 KD: (1) Mock DMSO *versus* Rab10 KD DMSO *p* < 0.0001, (2) Mock PLD inhibitors *versus* Rab10 KD DMSO *p* = 0.0069, (3) Mock washout *versus* Rab10 KD washout *p* < 0.0001, (4) Mock DMSO *versus* Mock PLD inhibitors *p* < 0.0001, (5) Mock DMSO *versus* washout *p* = 0.000304, (6) Mock PLD inhibitors *versus* Mock washout *p* < 0.0001, (7) Rab10 KD DMSO *versus* Rab10 KD PLD inhibitors *p* = 0.00036, (8) Rab10 KD DMSO *versus* Rab10 KD washout *p* = 0.17, (9) Rab10 KD PLD inhibitors *versus* Rab10 KD washout: *p* = 0.0034. For MICAL-L1 tubules in EHBP1 KD: (1) Mock DMSO *versus* EHBP1 KD DMSO *p* < 0.0001, (2) Mock PLD inhibitors *versus* EHBP1 KD DMSO *p* = 0.056, (3) Mock washout *versus* EHBP1 KD washout *p* = 0.41, (4) Mock DMSO *versus* Mock PLD inhibitors *p* < 0.0001, (5) Mock DMSO *versus* washout *p* = 0.000304, (6) Mock PLD inhibitors *versus* Mock washout *p* < 0.0001, (7) EHBP1 KD DMSO *versus* EHBP1 KD PLD inhibitors *p* < 0.0001, (8) EHBP1 KD DMSO *versus* EHBP1 KD washout *p* = 0.00073, (9) EHBP1 KD PLD inhibitors *versus* EHBP1 KD washout *p* = 0.00038. For Rab10 tubules in EHBP1 KD: (1) Mock DMSO *versus* EHBP1 KD DMSO *p* = 0.0056, (2) Mock PLD inhibitors *versus* EHBP1 KD DMSO *p* = 0.17, (3) Mock washout *versus* EHBP1 KD washout *p* = 0.26, (4) Mock DMSO *versus* Mock PLD inhibitors *p* = 0.00040, (5) Mock DMSO *versus* washout *p* = 0.029, (6) Mock PLD inhibitors *versus* Mock washout *p* = 0.0021, (7) EHBP1 KD DMSO *versus* EHBP1 KD PLD inhibitors *p* = 0.055, (8) EHBP1 KD DMSO *versus* EHBP1 KD washout *p* = 0.0019, (9) EHBP1 KD PLD inhibitors *versus* EHBP1 KD washout *p* = 0.00058. KD, knock-down.

We have previously demonstrated that phospholipase D (PLD) inhibitors may be used to acutely disrupt the TRE network within 30 min of treatment, likely by affecting levels of PA and potentially also PI(4,5)P2 (23). It is intriguing that once the PLD inhibitors have been washed out, the cells rapidly regenerate TREs, providing a unique system to address the roles of select proteins in the regeneration process (23). Although there is a rapid “overproduction” of TREs visualized after the washout of the PLD inhibitors, we have demonstrated that over several hours the TRE levels are restored to baseline, suggesting that this is due to a “lipid flux” upon washout (23). Capitalizing on this system, we next tested the role of Rab10 and EHBP1 on regeneration of TREs under these conditions. Initially, we first demonstrated that, in Mock-treated cells, MICAL-L1-marked TREs were dramatically abrogated upon PLD treatment but regenerated rapidly upon PLD washout (Fig. 6, *A*–*F*). Similarly, TREs marked by Rab10 were significantly impaired upon PLD treatment but recovered rapidly upon PLD washout (Fig. 6, *G*–*L*). These data further support the idea that MICAL-L1- and Rab10-TREs mostly mark the same tubular endosomes.

Using this system, we now addressed the role of Rab10 in TRE regeneration by knocking it down with siRNA. As demonstrated by immunoblot, Rab10 expression was significantly reduced with siRNA (Fig. 6*M*; quantified in Fig. 6*N*). We then calculated and compared the apparent MICAL-L1-TRE volume between Mock and Rab10 knock-down cells under control conditions (dimethyl sulfoxide [DMSO] treatment), PLD inhibitor treatment, and following inhibitor washout (Fig. 6*Q*). As expected, the apparent MICAL-L1-TRE volume was reduced by over 50% in DMSO-treated cells upon Rab10 knock-down compared with Mock-treated cells. PLD inhibitor treatment further reduced the apparent TRE volume levels in both Mock-treated and Rab10 knock-down cells. Most significantly, whereas the Mock-treated cells displayed a massive recovery in TRE regeneration upon washout, the Rab10 knock-down cells did not, and the TRE levels remained as low as the DMSO treatment baseline (Fig. 6*Q*). These data are consistent with the requirement of Rab10 for TRE biogenesis. We also tested the role of EHBP1 in TRE regeneration, since like Rab10, it appeared to be necessary for the biogenesis and/or maintenance of these endosomes. Accordingly, we used DMSO, PLD inhibitors, and washout of the inhibitors to compare apparent TRE volumes (marked by MICAL-L1 in Fig. 6*R*, or marked by Rab10 in Fig. 6*S*) in Mock-treated and EHBP1 knock-down cells. As expected, EHBP1 knock-down significantly reduced the apparent volumes of TREs marked by either MICAL-L1 (Fig. 6*R*) or Rab10 (Fig. 6*S*). Also as anticipated, PLD inhibitor treatment further reduced TRE levels of both Mock-treated and EHBP1 knock-down cells. However, somewhat surprisingly, upon PLD inhibitor washout the EHBP1 knock-down cells (Fig. 6, *O-P*) displayed TRE regeneration almost identical to that seen in Mock-treated cells (Fig. 6, *R*–*S*). These data suggest that Rab10 is a key protein involved in the biogenesis, maintenance, and regeneration of TREs. However, although EHBP1 appears to be required for TRE biogenesis and/or maintenance, unlike Rab10, EHBP1 appears to be expendable for TRE regeneration.

## Discussion

There is rising interest in the role of tubular endosomes as intermediates for endocytic membrane trafficking. Despite this attention, the molecular components of TREs remain incompletely characterized and the complex mechanisms of TRE biogenesis are only partly understood. In particular, whether the long, stable TREs marked by Rab10 (26) and those marked by MICAL-L1 and Syndapin2 (23) as well as EHD1 (33) are part of the same endosomal network, or whether they represent distinct TREs, remained unknown to date.

In our study, we show that MICAL-L1 and Rab10 are for the most part constituents of the same TRE structures, and a previous study has provided evidence that these two proteins may directly interact (34). Such structures, which may emanate either from SE or from the densely concentrated ERC, have been implicated in the recycling of various receptors to the plasma membrane (23, 26). However, some evidence exists that they may also regulate additional trafficking pathways (35, 36) and may partially overlap with tubules containing Rab8 and GRAF2 (37). Although the overlap measured between MICAL-L1 and Rab10 does not approach 100%, there are several possible reasons to explain this anomaly. First, although almost all the endogenously expressed MICAL-L1 localizes to TREs, a significant portion of the endogenous Rab10 staining is not localized to the TREs; this explains why the surface volume overlap of MICAL-L1 with Rab10 is significantly higher than that of Rab10 with MICAL-L1. Second, in a number of cases, one can visualize Rab10 and MICAL-L1 both localized to the same TREs, but they tend to segregate along the structure. This suggests that it is possible that both proteins coincide along the length of the TRE, but at differing concentrations, some of which may be below the threshold of detection. Moreover, we now demonstrate that TREs comprise both PA and PI(4,5)P2, with PA necessary for recruitment of MICAL-L1 and Syndapin2 and PI(4,5)P2 necessary for interactions with EHBP1 and/or Rab10.

In our initial examination of TRE biogenesis in 2013, fewer markers of these structures had been identified, with MICAL-L1, Syndapin2, and EHD1 being the primary known protein constituents (23). Based on the data available at that time demonstrating that Syndapin2 interacts with EHD1 (24, 38), the role of the former in membrane curvature (20, 21, 39), and our findings that the Syndapin SH3 domain interacts with MICAL-L1 via its proline rich regions (23), we arrived at a model for potential TRE biogenesis. We then proposed a scenario in which PA recruits MICAL-L1 and Syndapin2 to endosomal membranes and the subsequent interaction between the two proteins stabilizes them on the membranes. Once stabilized, we envisioned a role for Syndapin2 in the induction of membrane curvature via its BAR domain. However, the recent identification of Rab10 as a component of TREs (26) and its overlap with MICAL-L1/Syndapin2 on the same TRE membranes have led us to revise our TRE biogenesis model. Although we cannot entirely rule out the possibility that Rab10 has indirect effects on recycling endosomes, a key role in the biogenesis of TREs is consistent with our data and those of Etoh and Fukuda (26), and consistent with the known localization and function of Rab10 in mammalian cells (40) and invertebrates (31, 41).

Based on the localization of Rab10 to TREs, we now find that, surprisingly, TRE biogenesis occurs even in the absence of MICAL-L1 and/or Syndapin2. Indeed, as demonstrated in Fig. 3, *N–O*, not only do TRE volumes not decrease upon MICAL-L1 or Syndapin2 depletion, but also the mean TRE volume actually shows a small but significant increase. This suggests a potential role for MICAL-L1 and Syndapin2 in TRE fission, rather than biogenesis, a role that fits previous studies describing a role for Syndapin proteins in vesicle generation (20, 42) and suggests that they may coordinate fission with EHD1 (15, 43, 44, 45, 46, 47).

**Figure 7:**
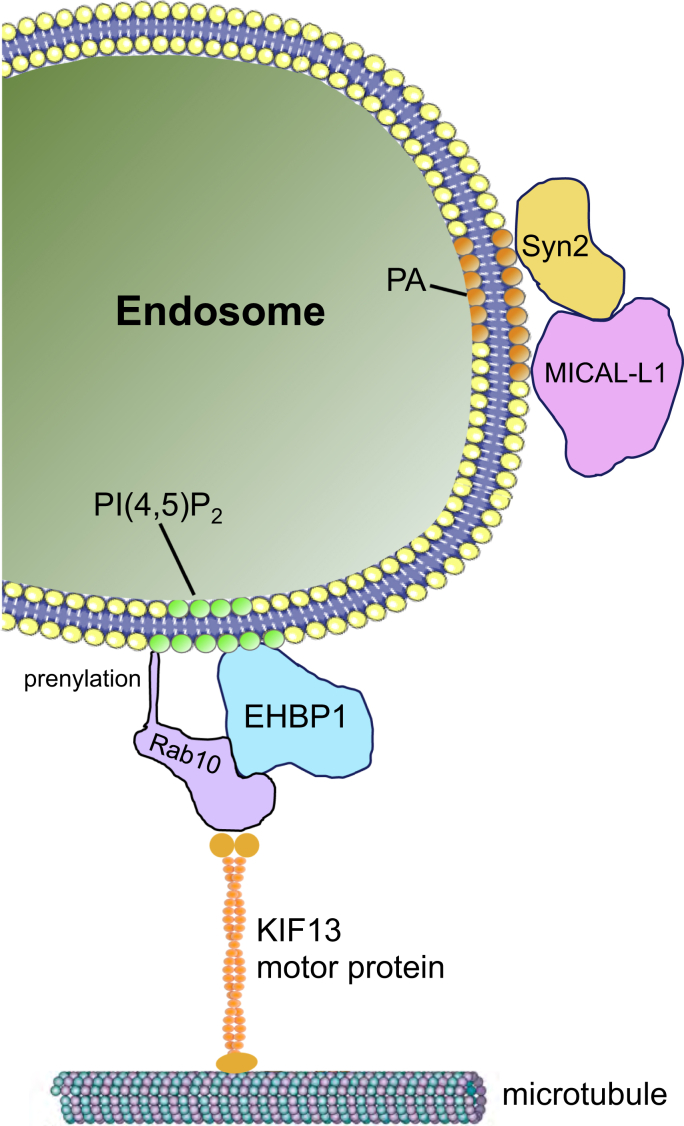
**Schematic model for the biogenesis of tubular recycling endosomes.** EHBP1 helps link PI(4,5)P2-containing endosomes to Rab10 and KIF13 motor proteins, which may serve as the driving force to pull the endosomal membranes along microtubules and thus generate tubular recycling endosomes.

Although Rab10 and EHBP1 are required for TRE biogenesis (or at least maintenance of TRE), only the presence of Rab10 is necessary for acute regeneration of TREs following PA depletion. EHBP1 has been implicated in fission of tubular endosomes (30, 41), a function consistent with its interaction with EHD proteins (27, 32, 48). Despite these findings, EHBP1 knock-down in this study supports a general role in TRE biogenesis, but somewhat intriguingly, not regeneration of TRE following washout of PLD inhibitors. How do we envision the mechanisms for TRE biogenesis in view of these new data? The current data are consistent with a model in which Rab10 plays a major role in TRE biogenesis by both binding to motor proteins such as KIF13A/B (26, 49) and by binding to endosomes. In this manner, Rab10 would provide the key link between the endosome and the microtubule tracks, thus supporting the stretching and tubulation of the endosomal membrane upon motor protein movement (see model, Fig. 7). How would Rab10 link to endosomes? Various studies promote the idea that EHBP1 serves as an important interaction partner for Rab10 on the endosomal membrane, including TRE membranes (30, 31, 41). Moreover, EHBP1 localizes to endosomes through a direct interaction with PI(4,5)P2 (31), suggesting a role for this lipid in recruiting EHBP1 and linking endosomes to motor proteins to facilitate tubulation. One finding not entirely consistent with this model was that EHBP1 appeared to be expendable for TRE regeneration after washout of PLD inhibitors. However, we rationalize that such regeneration conditions may not precisely mimic normal TRE biogenesis, and Rab10, like most Rab proteins, undergoes prenylation (50) and may be capable of directly interacting with endosomal membranes via its prenyl group even in the absence of EHBP1. Overall, our study defines the key lipid and protein constituents of TREs, highlights the role of Rab10 in TRE biogenesis, and offers a revised model to explain the mechanism of TRE biogenesis.

## Experimental procedures

### Cell lines

The HeLa cervical cancer cell line was obtained from the american type culture collection and grown in Dulbecco's modified Eagle's medium (high-glucose) containing 10% FBS, 1× penicillin–streptomycin (Invitrogen), and 2 mM glutamine. The MEFs derived from Rab10 knockout (KO) embryos were previously described (32) and grown in RPMI media containing 20% fetal bovine serum (FBS), 1× penicillin–streptomycin (Invitrogen), and 2 mM glutamine. All cell lines were routinely tested for *Mycoplasma* infection.

### Antibodies and reagents

The following antibodies were used: anti-MICAL-L1 (ab220648, Abcam, for immunoblots; H00085377, Abnova, for immunofluorescence), anti-Rab10 (ab237703, Abcam, for immunoblots and immunofluorescence), anti-EHBP1 (NBP1-93615, Novus, for immunoblots), anti-Syndapin2/Pacsin2 (SAB1300127, Sigma, for immunoblots), anti-EEA1 (NBP1-05962, Novus for immunoblots), anti-pPKCα (06-822, Millipore, for immunoblots), anti-GAPDH-horseradish peroxidase (HRP) (HRP-60004, Proteintech, for immunoblots), mouse anti-rabbit IgG light chain–HRP (211-032-171, Jackson, for immunoblots), Alexa Fluor 568–conjugated goat anti-rabbit (A11036, Molecular Probes, for immunofluorescences), Alexa Fluor 568–conjugated goat anti-mouse (A11031, Molecular Probes, for immunofluorescence), and Alexa Fluor 488–conjugated donkey anti-mouse (A21202, Molecular Probes, for immunofluorescence). The PLD inhibitors CAY 10593 (also known as VU0155069) and CAY 10594 were purchased from Cayman Chemical Co and were typically used at 50 μM for 30 min at 37 °C.

### Immunoblotting

Cells were washed twice in ice-cold phosphate-buffered saline (PBS) and were then scraped off plates with a rubber policeman into ice-cold lysis buffer (50 mM Tris, pH 7.4, 100 mM NaCl, 0.5% TX-100, 1× protease cocktail inhibitor [Millipore]). Protein levels of postnuclear lysates were quantified using the Bradford assay (Bio-Rad) for equal protein level loading. For immunoblotting, 20 to 30 μg of protein per lysate was separated by SDS-PAGE. Proteins were transferred onto nitrocellulose membranes and blocked for 30 min at room temperature in PBS with 0.3% Tween (PBST) plus 5% nonfat dry milk. The membranes were then incubated overnight at 4 °C or for 1 h at room temperature with primary antibodies diluted in PBST. Membranes were then washed three times with PBST and incubated at room temperature with appropriate secondary antibodies in PBST for 30 min. The membranes were then washed again three times with PBST, before being subjected to enhanced chemiluminescence.

### Fractionation to membrane and cytosolic fractions

HeLa cells plated on 10-cm plates were treated with EHBP1 siRNA or mock transfected (48 h) using Lipofectamine RNAiMAX according to the manufacturer's instructions. Cells were then resuspended with Cellstripper (Corning #25-056-CI) and centrifuged. Cell pellets were resuspended with Homogenization Buffer (25 mM Hepes, 100 mM NaCl, 1 mM EDTA) containing protease inhibitors (Calbiochem #539131) and homogenized on ice. Homogenates were centrifuged for 1 h at 4 °C at 100,000*g*. Supernatants (cytosolic fractions) and pellets (membrane fractions) were boiled in 4× Laemmli sample buffer, and proteins samples were separated and visualized by SDS-PAGE and immunoblotting.

### Quantification of immunoblots

The adjusted relative density of the immunoblots was measured in Fiji ImageJ based on the method in the following protocol: https://imagej.nih.gov/ij/docs/menus/analyze.html#gels. Statistical significance was calculated using a Student's *t*-test with the Vassarstats program (http://www.vassarstats.net).

### siRNA treatment

HeLa cells were grown either on coverslips or 35-mm culture dishes for 24 h in Dulbecco's modified Eagle's medium containing 10% FBS with 2 mM L-glutamine and 1× units/ml penicillin/streptomycin. The cells were then subjected to siRNA treatment with human MICAL-L1 (ON-TARGETplus SMARTPool, L-015102-01-0010), Syndapin-2 (ON-TARGETplus SMARTPool, L-019666-02-0010), Rab10 (ON-TARGETplus SMARTPool, L-010823-00-0010), or EHBP1 (TARGETplus SMARTPool, L-014061-01-0010) oligonucleotides from Dharmacon for 48 h at 37 °C using Dharmafect (Dharmacon, T-2001-03), following the manufacturer's protocol.

### Transfection

MEFs were cultured on coverslips and transfected with either green fluorescent protein or red fluorescent protein-Rab10 using GeneExpresso Max transfection reagent (Excellgen) for 48 h.

### Plasmids

Red fluorescent protein-Rab10 was a gift from Dr Barth Grant. The PLCδ1 PH domain (marker for PI(4,5)P2) and MICAL-L1 CC (marker for PA) have been described previously (23, 51).

### Immunofluorescence

HeLa or MEF cells were treated as indicated in the text and then fixed with 4% paraformaldehyde in PBS for 10 min at room temperature. Cells were then rinsed three times in PBS and incubated with primary antibody in PBS containing 0.5% bovine serum albumin and 0.2% saponin for 1 h at room temperature. Cells were then washed three times in PBS and incubated with the appropriate fluorochrome-conjugated secondary antibodies diluted in PBS containing 0.5% bovine serum albumin and 0.2% saponin for 30 min. Cells were then washed three times in PBS and mounted in Fluoromount. Z-Stack confocal imaging was performed using a Zeiss LSM 800 confocal microscope with a 63×/1.4 numerical aperture oil objective, and more than 10 cells from three independent experiments were processed using the IMARIS software.

### Image processing using the IMARIS

Z-Sections of images (8–12 slices) acquired from the confocal microscope were imported into IMARIS x64 9.1.2 software (Bitplane AG, Zurich, Switzerland) coupled with custom MAT-LAB (2009 and 2014) programming for 3D surface rendering and quantitative analysis, as indicated. Briefly, the image display was adjusted for both of the channels (MICAL-L1, green; Rab10, red), and the rendering quality was set to 100%. Surfaces were created by selecting the source channel and smooth surface detail set at 0.198 m. Background subtraction was set to 0.743 m, and the threshold was reduced for surfaces to fully cover all voxels. The surface area and volume of the surfaces generated were quantified by IMARIS for both of the channels, and the values were exported into Excel for graphical and statistical analysis. To quantify apparent surface overlap volume between two surfaces (MICAL-L1 and Rab10), the IMARIS XT bundle Kiss and Run was first integrated with MATLAB and launched in IMARIS. The 3D surface-reconstructed images were then processed for Kiss and Run analysis using the surface–surface overlap module, which uses a surface mask for the target and tracks the surface and determines the overlap for each surface independently. This particular Xtension program analyzes contact events between surfaces that are defined by having at least one overlapping voxel. The volume of overlap for each surface was then quantified and exported to Excel for further analysis. The mean and the SE of the mean were calculated from data obtained from three independent experiments with at least 10 images taken per treatment. Statistical significance was calculated using a Student's *t*-test with the Vassarstats program (http://www.vassarstats.net). Snapshots and/or videos were obtained from the IMARIS program and used as representative images.

### Structured illumination microscopy and data processing

Structured illumination microscopy (SIM) images were collected with a Zeiss Elyra PS.1 illumination system (Carl Zeiss) using a 63× oil objective with numerical aperture of 1.4. Two laser lines were used in acquisition of images: 488 and 568 nm. Three orientation angles of the excitation grid were acquired for each Z-plane, with Z spacing of 110 nm between planes. SIM processing was performed with the SIM module of the Zen Black software (Carl Zeiss). The processed SIM images were then exported in TIF format.

## Data availability

All data are contained within the article.

## Conflict of interest

The authors declare that they have no conflicts of interest with the contents of this article.

## References

[1] Conner S.D., Schmid S.L. (2003). Regulated portals of entry into the cell. Nature.

[2] Naslavsky N., Caplan S. (2018). The enigmatic endosome - sorting the ins and outs of endocytic trafficking. J. Cell Sci..

[3] Hsu J.W., Bai M., Li K., Yang J.S., Chu N., Cole P.A., Eck M.J., Li J., Hsu V.W. (2020). The protein kinase Akt acts as a coat adaptor in endocytic recycling. Nat. Cell Biol..

[4] Bai M., Pang X., Lou J., Zhou Q., Zhang K., Ma J., Li J., Sun F., Hsu V.W. (2012). Mechanistic insights into regulated cargo binding by ACAP1 protein. J. Biol. Chem..

[5] Steinberg F., Heesom K.J., Bass M.D., Cullen P.J. (2012). SNX17 protects integrins from degradation by sorting between lysosomal and recycling pathways. J. Cell Biol..

[6] Clairfeuille T., Mas C., Chan A.S., Yang Z., Tello-Lafoz M., Chandra M., Widagdo J., Kerr M.C., Paul B., Merida I., Teasdale R.D., Pavlos N.J., Anggono V., Collins B.M. (2016). A molecular code for endosomal recycling of phosphorylated cargos by the SNX27-retromer complex. Nat. Struct. Mol. Biol..

[7] van der Sluijs P., Hull M., Webster P., Male P., Goud B., Mellman I. (1992). The small GTP-binding protein rab4 controls an early sorting event on the endocytic pathway. Cell.

[8] Ullrich O., Reinsch S., Urbe S., Zerial M., Parton R.G. (1996). Rab11 regulates recycling through the pericentriolar recycling endosome. J. Cell Biol..

[9] Weigert R., Yeung A.C., Li J., Donaldson J.G. (2004). Rab22a regulates the recycling of membrane proteins internalized independently of clathrin. Mol. Biol. Cell.

[10] Donaldson J.G. (2003). Multiple roles for Arf6: sorting, structuring, and signaling at the plasma membrane. J. Biol. Chem..

[11] Maxfield F.R., McGraw T.E. (2004). Endocytic recycling. Nat. Rev. Mol. Cell Biol..

[12] Frost A., Unger V.M., De Camilli P. (2009). The BAR domain superfamily: membrane-molding macromolecules. Cell.

[13] Gallop J.L., McMahon H.T. (2005). BAR domains and membrane curvature: bringing your curves to the BAR. Biochem. Soc. Symp..

[14] van Weering J.R., Verkade P., Cullen P.J. (2010). SNX-BAR proteins in phosphoinositide-mediated, tubular-based endosomal sorting. Semin. Cell Dev. Biol..

[15] Cai B., Xie S., Caplan S., Naslavsky N. (2014). GRAF1 forms a complex with MICAL-L1 and EHD1 to cooperate in tubular recycling endosome vesiculation. Front. Cell Dev. Biol..

[16] Giridharan S.S., Cai B., Naslavsky N., Caplan S. (2012). Trafficking cascades mediated by Rab35 and its membrane hub effector, MICAL-L1. Commun. Integr. Biol..

[17] Rahajeng J., Giridharan S.S., Cai B., Naslavsky N., Caplan S. (2012). MICAL-L1 is a tubular endosomal membrane hub that connects Rab35 and Arf6 with Rab8a. Traffic.

[18] Sharma M., Giridharan S.S., Rahajeng J., Caplan S., Naslavsky N. (2010). MICAL-L1: an unusual Rab effector that links EHD1 to tubular recycling endosomes. Commun. Integr. Biol..

[19] Sharma M., Giridharan S.S., Rahajeng J., Naslavsky N., Caplan S. (2009). MICAL-L1 links EHD1 to tubular recycling endosomes and regulates receptor recycling. Mol. Biol. Cell.

[20] Kessels M.M., Qualmann B. (2006). Syndapin oligomers interconnect the machineries for endocytic vesicle formation and actin polymerization. J. Biol. Chem..

[21] Senju Y., Itoh Y., Takano K., Hamada S., Suetsugu S. (2011). Essential role of PACSIN2/syndapin-II in caveolae membrane sculpting. J. Cell Sci..

[22] Wang Q., Navarro M.V., Peng G., Molinelli E., Goh S.L., Judson B.L., Rajashankar K.R., Sondermann H. (2009). Molecular mechanism of membrane constriction and tubulation mediated by the F-BAR protein Pacsin/Syndapin. Proc. Natl. Acad. Sci. U. S. A..

[23] Giridharan S.S., Cai B., Vitale N., Naslavsky N., Caplan S. (2013). Cooperation of MICAL-L1, syndapin2, and phosphatidic acid in tubular recycling endosome biogenesis. Mol. Biol. Cell.

[24] Braun A., Pinyol R., Dahlhaus R., Koch D., Fonarev P., Grant B.D., Kessels M.M., Qualmann B. (2005). EHD proteins associate with syndapin I and II and such interactions play a crucial role in endosomal recycling. Mol. Biol. Cell.

[25] Xie S., Bahl K., Reinecke J.B., Hammond G.R., Naslavsky N., Caplan S. (2016). The endocytic recycling compartment maintains cargo segregation acquired upon exit from the sorting endosome. Mol. Biol. Cell.

[26] Etoh K., Fukuda M. (2019). Rab10 regulates tubular endosome formation through KIF13A and KIF13B motors. J. Cell Sci..

[27] Guilherme A., Soriano N.A., Furcinitti P.S., Czech M.P. (2004). Role of EHD1 and EHBP1 in perinuclear sorting and insulin-regulated GLUT4 recycling in 3T3-L1 adipocytes. J. Biol. Chem..

[28] Rebecchi M., Boguslavsky V., Boguslavsky L., McLaughlin S. (1992). Phosphoinositide-specific phospholipase C-delta 1: effect of monolayer surface pressure and electrostatic surface potentials on activity. Biochemistry.

[29] Rebecchi M., Peterson A., McLaughlin S. (1992). Phosphoinositide-specific phospholipase C-delta 1 binds with high affinity to phospholipid vesicles containing phosphatidylinositol 4,5-bisphosphate. Biochemistry.

[30] Gao J., Zhao L., Luo Q., Liu S., Lin Z., Wang P., Fu X., Chen J., Zhang H., Lin L., Shi A. (2020). An EHBP-1-SID-3-DYN-1 axis promotes membranous tubule fission during endocytic recycling. PLoS Genet..

[31] Wang P., Liu H., Wang Y., Liu O., Zhang J., Gleason A., Yang Z., Wang H., Shi A., Grant B.D. (2016). RAB-10 promotes EHBP-1 bridging of filamentous actin and tubular recycling endosomes. PLoS Genet..

[32] Li Z., Schulze R.J., Weller S.G., Krueger E.W., Schott M.B., Zhang X., Casey C.A., Liu J., Stockli J., James D.E., McNiven M.A. (2016). A novel Rab10-EHBP1-EHD2 complex essential for the autophagic engulfment of lipid droplets. Sci. Adv..

[33] Caplan S., Naslavsky N., Hartnell L.M., Lodge R., Polishchuk R.S., Donaldson J.G., Bonifacino J.S. (2002). A tubular EHD1-containing compartment involved in the recycling of major histocompatibility complex class I molecules to the plasma membrane. EMBO J..

[34] Fukuda M., Kanno E., Ishibashi K., Itoh T. (2008). Large scale screening for novel rab effectors reveals unexpected broad Rab binding specificity. Mol. Cell Proteomics.

[35] McKenzie J.E., Raisley B., Zhou X., Naslavsky N., Taguchi T., Caplan S., Sheff D. (2012). Retromer guides STxB and CD8-M6PR from early to recycling endosomes, EHD1 guides STxB from recycling endosome to Golgi. Traffic.

[36] Abou-Zeid N., Pandjaitan R., Sengmanivong L., David V., Le Pavec G., Salamero J., Zahraoui A. (2011). MICAL-like1 mediates epidermal growth factor receptor endocytosis. Mol. Biol. Cell.

[37] Lucken-Ardjomande Hasler S., Vallis Y., Pasche M., McMahon H.T. (2020). GRAF2, WDR44, and MICAL1 mediate Rab8/10/11-dependent export of E-cadherin, MMP14, and CFTR DeltaF508. J. Cell Biol..

[38] Xu Y., Shi H., Wei S., Wong S.H., Hong W. (2004). Mutually exclusive interactions of EHD1 with GS32 and syndapin II. Mol. Membr. Biol..

[39] Quan A., Xue J., Wielens J., Smillie K.J., Anggono V., Parker M.W., Cousin M.A., Graham M.E., Robinson P.J. (2012). Phosphorylation of syndapin I F-BAR domain at two helix-capping motifs regulates membrane tubulation. Proc. Natl. Acad. Sci. U. S. A..

[40] Chua C.E.L., Tang B.L. (2018). Rab 10-a traffic controller in multiple cellular pathways and locations. J. Cell Physiol..

[41] Shi A., Chen C.C., Banerjee R., Glodowski D., Audhya A., Rongo C., Grant B.D. (2010). EHBP-1 functions with RAB-10 during endocytic recycling in Caenorhabditis elegans. Mol. Biol. Cell.

[42] Koch D., Spiwoks-Becker I., Sabanov V., Sinning A., Dugladze T., Stellmacher A., Ahuja R., Grimm J., Schuler S., Muller A., Angenstein F., Ahmed T., Diesler A., Moser M., Tom Dieck S. (2011). Proper synaptic vesicle formation and neuronal network activity critically rely on syndapin I. EMBO J..

[43] Cai B., Caplan S., Naslavsky N. (2012). cPLA2alpha and EHD1 interact and regulate the vesiculation of cholesterol-rich, GPI-anchored, protein-containing endosomes. Mol. Biol. Cell.

[44] Cai B., Giridharan S.S., Zhang J., Saxena S., Bahl K., Schmidt J.A., Sorgen P.L., Guo W., Naslavsky N., Caplan S. (2013). Differential roles of C-terminal Eps15 homology domain proteins as vesiculators and tubulators of recycling endosomes. J. Biol. Chem..

[45] Dhawan K., Naslavsky N., Caplan S. (2020). Sorting nexin 17 (SNX17) links endosomal sorting to Eps15 homology domain protein 1 (EHD1)-mediated fission machinery. J. Biol. Chem..

[46] Deo R., Kushwah M.S., Kamerkar S.C., Kadam N.Y., Dar S., Babu K., Srivastava A., Pucadyil T.J. (2018). ATP-dependent membrane remodeling links EHD1 functions to endocytic recycling. Nat. Commun..

[47] Kamerkar S.C., Roy K., Bhattacharyya S., Pucadyil T.J. (2019). A screen for membrane fission catalysts identifies the ATPase EHD1. Biochemistry.

[48] Guilherme A., Soriano N.A., Bose S., Holik J., Bose A., Pomerleau D.P., Furcinitti P., Leszyk J., Corvera S., Czech M.P. (2004). EHD2 and the novel EH domain binding protein EHBP1 couple endocytosis to the actin cytoskeleton. J. Biol. Chem..

[49] Delevoye C., Miserey-Lenkei S., Montagnac G., Gilles-Marsens F., Paul-Gilloteaux P., Giordano F., Waharte F., Marks M.S., Goud B., Raposo G. (2014). Recycling endosome tubule morphogenesis from sorting endosomes requires the kinesin motor KIF13A. Cell Rep..

[50] Kohnke M., Delon C., Hastie M.L., Nguyen U.T., Wu Y.W., Waldmann H., Goody R.S., Gorman J.J., Alexandrov K. (2013). Rab GTPase prenylation hierarchy and its potential role in choroideremia disease. PLoS One.

[51] Varnai P., Balla T. (1998). Visualization of phosphoinositides that bind pleckstrin homology domains: calcium- and agonist-induced dynamic changes and relationship to myo-[3H]inositol-labeled phosphoinositide pools. J. Cell Biol..

