# Supplementary material for: Defining the protein and lipid constituents of tubular recycling endosomes
Source: J Biol Chem. 2021 Jan 28;296:100190. doi: 10.1074/jbc.RA120.015992 (PMC7948492; doi:10.1074/jbc.RA120.015992)
Supplement: Figures S1 and S2 [file mmc2.pdf]

Supplemental Fig. 1

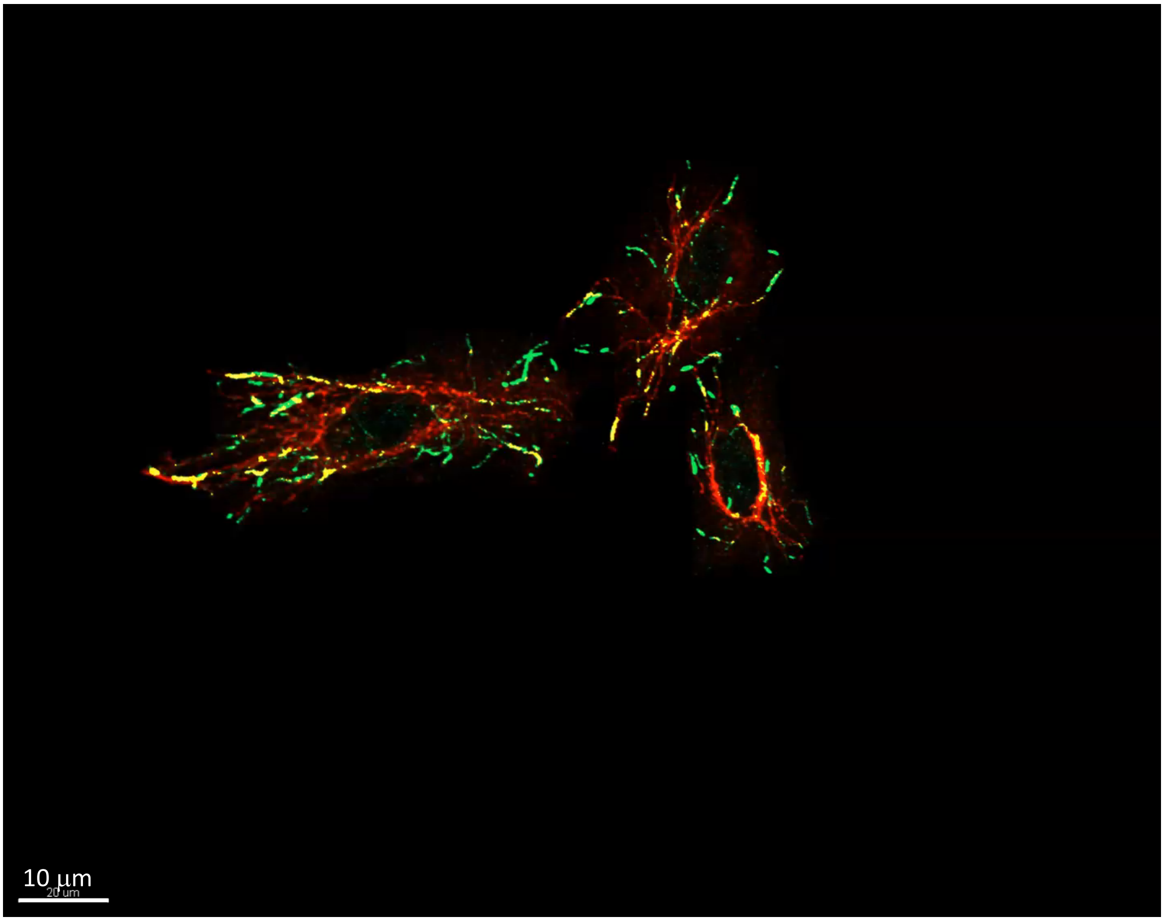

Supplemental Figure 1: MICAL-L1 and Rab10 reside on the same tubular recycling endosomes. HeLa cells were cultured on cover-slides, fixed and immunostained with antibodies against endogenous MICAL-L1 (*A*, green) and Rab10 (*B*, red). A series of serial section were obtained and the representative movie is a 3D reconstitution.

Supplemental Fig. 2

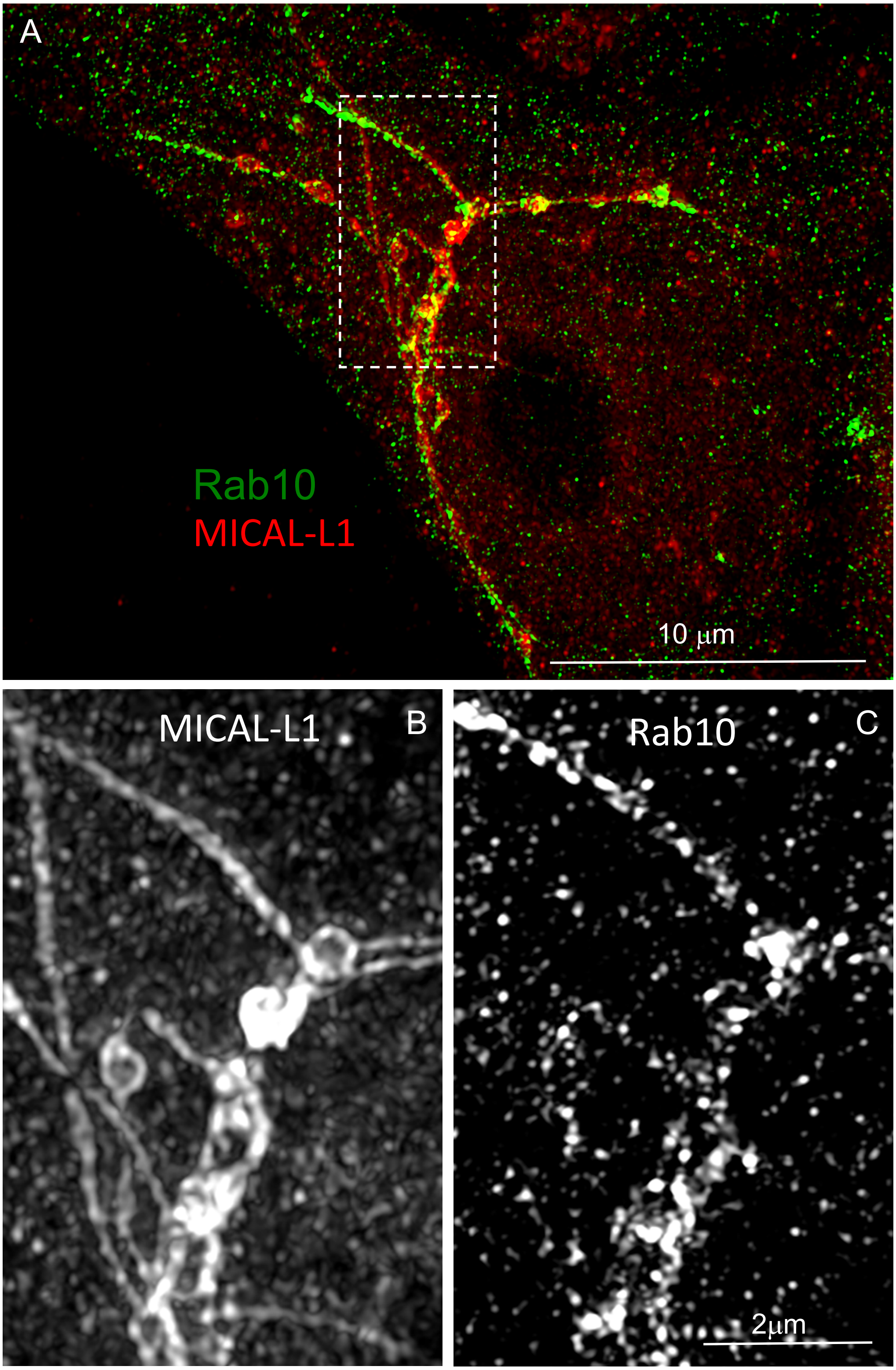

Supplemental Figure 2: super-resolution Structured Illumination Imaging of MIAL-L1 and Rab10 on tubular endosomes. HeLa cells were cultured on cover-slides, fixed and immunostained with antibodies against endogenous MICAL-L1 (*A*, red and B) and Rab10 (*A*, green and C). Serial z-sections were obtained by super-resolution Structured Illumination Imaging. Bar, 10 mm.
